# Gastrointestinal Complaints and Correlations with Self-Reported Macronutrient Intake in Independent Groups of (Ultra)Marathon Runners Competing at Different Distances

**DOI:** 10.3390/sports7060140

**Published:** 2019-06-07

**Authors:** Daan Hoogervorst, Nancy van der Burg, Joline J. Versteegen, Karin J. Lambrechtse, Martijn I. Redegeld, Larissa A. J. Cornelissen, Floris C. Wardenaar

**Affiliations:** 1 Sports and Exercise Nutrition, Institute for Sports and Exercise, HAN University of Applied Sciences, Nijmegen 6525, The Netherlands; hoogervorstdaan@gmail.com (D.H.); vanderburgnancy@gmail.com (N.v.d.B.); joline.versteegen@gmail.com (J.J.V.); info@karinlambrechtse.nl (K.J.L.); martijn.redegeld@kuleuven.be (M.I.R.); lcornelissen@hotmail.nl (L.A.J.C.); 2 Nutrition & Performance, Team Jumbo-Visma, ‘s-Herthogenbosch 5222, The Netherlands; 3 Global Nutrition Development, Friesland Campina, Amersfoort 3818, The Netherlands; 4 Bakala Academy Athletic Performance Center, Katholieke Universiteit Leuven, Leuven 3000, Belgium; 5 College of Health Solutions, Arizona State University, Phoenix, AZ 85004, USA

**Keywords:** running, sports nutrition, GI complaints, marathon, Food and Fluid Exercise Questionnaire (FFEQ), ultrarunning, endurance exercise

## Abstract

This study investigated the differences in gastrointestinal (GI) and exercise related complaints between groups of runners competing at different distances using web-based questionnaires. Total (severe) complaints were reported by 89.3% (49.7%) of the runners during the race vs. 70.6% (29.4%) after the race. Significant differences between groups were described for marathon (n = 98) and 60 km (n = 43) runners. During competition, runners reported the urge to urinate (47.7%), muscle cramps (43.6%) and belching (43.6%). The prevalence of bloating, flatulence, side ache and dizziness differed between distances (*p* < 0.02). There were small to moderate negative correlations between food and fluid intake and GI complaints. After competition (12 h), 70.6% of participants reported complaints, with muscle cramps (47.6%), flatulence (19.0%) and bloating (12.7%) being the most prevalent. Prevalence of belching, nausea, stomach cramps and muscle cramps differed between race distances (*p* < 0.04). There were small to high positive correlations between complaints during and after competition (*p* < 0.05). In conclusion, runners of all distances reported a high prevalence of GI and other exercise related complaints. There were some small differences in reporting type and severity of complaints between distances. Results showed small to strong correlations between complaints during and after competition and with nutrient intake, without a clear similar pattern for all distances.

## 1. Introduction

Participation in endurance events such as marathons and ultramarathons has been associated with a high incidence of gastrointestinal (GI) complaints [1,2]. These complaints include upper GI complaints such as belching, bloating, reflux and nausea, lower GI complaints such as flatulence, side ache and urge to defecate, and other exercise related complaints such as the urge to urinate and muscle cramps [3,4]. Although it seems reasonable to suggest that GI complaints occur during running as a result of gut damage due to mechanical trauma with injury of the intestinal mucosa by splanchnic hypoperfusion leading to intestinal ischemia, there is a lack of evidence showing a direct relationship between GI complaints and gut damage [5]. While a clear relationship between intestinal damage and GI complaints is not evident, it is estimated that prevalence and severity of GI complaints can be related to distance, fitness level, the excessive intake and amount of food and fluid, exercise intensity, environmental conditions, gender, the history of GI complaints, or a combination of all these factors [6,7]. It has been reported that the severity of GI problems increases with exercise time [8], which may be reflected in field observations of different running distances. GI complaints varied from 30–50% in marathon running [9], 83% at 60 km in a previous study reported by our lab [3], and up to 96% in a 161 km ultramarathon [10]. 

Adequate intake of food and fluid has been shown to decrease the chance of developing GI complaints [6,11,12]. Based on general recommendations, athletes exercising for more than three hours (e.g., during a (ultra)marathon) are advised to consume 60–90 grams of carbohydrate (CHO) per hour [13]. An insufficient CHO supply leads to faster skeletal muscle fatigue, thus reducing performance [14,15,16]. On the other hand, well-trained ultrarunners may finish a race with only half the amount of the recommended CHO intake [4,17]. In addition, maintaining an adequate fluid balance may also help to reduce GI complaints, as both dehydration and overhydration can lead to hyponatremia, splanchnic hypoperfusion and changes in gastrointestinal function and complaints [18]. Fluid recommendations may differ for marathon distance and distances exceeding a double marathon, as body weight changes may be not appropriate for a hydration status assessment due to the body mass loss associated with endogenous substrate loss and associated water loss [19]. Therefore, the general recommendation for all distances is to drink to thirst but fluid loss should not exceed 2–3% of total body weight [13,20]. Based on ultrarunning event registrations, fluid intake probably should range between 354–765 mL/h [3,4,10,21,22,23], depending on the athlete’s individual fluid loss [24]. 

Endurance athletes who find it difficult to meet these recommendations are advised to train their food and fluid strategy as part of their regular practice sessions [11]. Intake of food and fluid containing high amounts of fat and dietary fiber during exercise has been associated with GI complaints such as vomiting, stomach cramps and intestinal cramps [6,12]. Thus, avoiding intake of foods with a high fiber and fat content before and during exercise has been recommended to reduce GI complaints such as vomiting and intestinal cramps [24,25]. While individual needs often vary, when taken as a group, endurance and ultra-endurance runners often do not meet the higher end of sports nutrition recommendations for both CHOs and fluids [3,21,22,26].

In addition to commercially available sport nutrition products, alternative products (fruit, bars and sandwiches) may help athletes meet sport nutrition recommendations [4,21]. Sport foods may be more convenient because they are more concentrated, deliver a higher amount of nutrients, and are easier to use during exercise. However, use of these commercial products may also indirectly cause more GI complaints because of their high CHO, protein and/or electrolyte concentration, and/or their high acidity [12,27]. Anecdotally, ultra-runners use more alternative foods and beverages such as chocolate milk, soup, trail mix and beer [4,22]. These choices may help to combat the limited variety of sports foods, and provide more appealing tastes after consuming sweet (carbohydrate-rich) products during a race. However, these alternative foods and beverages may be high in fat and fiber. All of these nutritional elements are associated with a higher chance of developing GI complaints [24]. 

The common view is that the prevalence and severity of GI complaints are related to distance, resulting in both a larger number and higher intensity of complaints when athletes cover longer distances [6]. In particular, serious GI complaints are most often expressed when runners exceed the marathon distance [2]. The objective of this study was to investigate differences between independent groups of runners competing at marathon, 60 km and 120 km distance for self-reported GI and exercise related complaints during and after the race. As well, we describe correlations between complaints during and after the race, and correlations between complaints and nutritional intake for all distances during competition.

## 2. Materials and Methods

### 2.1. Study Design

This cross-sectional study collected detailed information using three web-based questionnaires on Qualtrics (The Qualtrics Research Suite, 2013. Provo, UT) about GI complaints and food and fluid intake in marathon and ultramarathon athletes. The study was approved by the Ethical Advisory Board of the HAN University of Applied Sciences (EACO 63.03/17) and performed in accordance with the Declaration of Helsinki. The questionnaires included a screening questionnaire, Food and Fluid Exercise questionnaire (FFEQ) and an additional post-race complaints questionnaire. The screening questionnaire had to be completed before starting the race and was available in a web-based form the week before the race and as a paper version the day of the race. Runners received an e-mail during the race that contained the FFEQ, which had to be completed before midnight after finishing the race. All runners who successfully completed the FFEQ in time received the post-race complaints questionnaire. The post-race complaints questionnaire had to be completed before midnight on the day after the race. 

### 2.2. Participants and Recruitment

Runners were recruited by e-mail, online ads and newsletters from each specific race. After showing interest, all runners were informed about the study design and informed consent was obtained. A total of 252 screening questionnaires were collected (177 from the marathon, 64 from the 60 km and 11 from the 120 km) with 149 runners completing the screening questionnaire and FFEQ (55.4%) and 126 runners completing all three questionnaires (50%). This study contains data for eight different running events in the Netherlands, i.e., six marathons (42.195 km) and two ultramarathons (60 km and 120 km). All events took place between September 2016 and March 2017.

### 2.3. Procedures

#### 2.3.1. Screening Questionnaire 

The web-based screening questionnaire asked about personal characteristics (gender, age, height, weight) and other factors (lifestyle, running history and general health) and previous GI and other exercise related complaints. For this study, only personal characteristics were reported. 

#### 2.3.2. Food and Fluid Exercise Questionnaire (FFEQ) 

**Figure 1:**
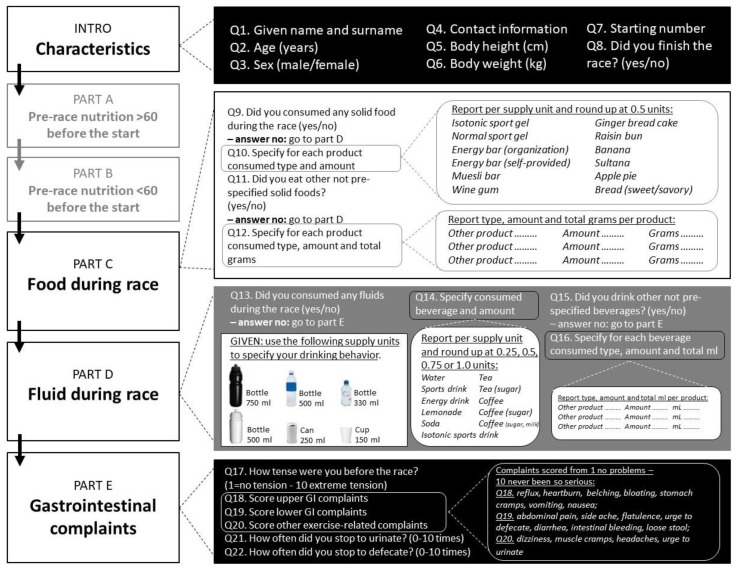
Overview of the Food and Fluid Exercise Questionnaire (FFEQ).

The FFEQ, an adapted version of previous work by Pfeiffer et al. (2012), contains separate questions about food and fluid intake pre-exercise (an hour before starting) and during exercise [1]. No data is available about the validity and/or reliability of the original and current versions of the questionnaire. The FFEQ consists of an introduction and five different parts. For the purpose of this study, the characteristics obtained in the introduction, the information about food and fluid intake during exercise in part C and D, and the data about GI complaints in part E were reported (Figure 1). 

Part C contained pre-specified food options that needed to be provided per whole or half serving, such as isotonic sports gel, energy gel, different types of energy bars or pastries (commercially available or homemade, i.e., muesli bar, gingerbread (slice)), banana, chewables such as wine gums (pieces), bread with sweet filling, and bread with savory filling. As well, part D included the following fluid options: water (750/500/330/150 mL), sports drink (750/500/330/150 mL), isotonic sports drink (750/500/330/150 mL), lemonade (750/500/330/150 mL), can of soda, can of energy drink, cup of tea or cup of coffee. After this, runners were asked in a yes/no format if they consumed any other products during the race. If yes, participants were able to list up to four different product options, identifying type (and brand if available) total number and total grams or milliliters (if known). To calculate energy and macronutrient intakes, product label declarations or the Dutch food composition database version 2016/5.0 were used [28]. Total product consumption (grams or milliliters of fluid) was calculated using standardized reference products. The most significant change to the original Pfeiffer questionnaire was the addition of photographic examples of different sizes and bottles at each drinking section. This helped athletes to identify the size of the fluid source, allowing the research team to accurately estimate the actual amount of fluid consumed. The organizations hosting the running events provided regular aid stations and also allowed runners to provide their own food and fluid items during the race. Part E of the FFEQ contained the original translated list of GI and exercise related complaints based on the questionnaire of Pfeiffer et al., which to the best of our knowledge has not been previously validated. This section contained an extensive list of complaints during exercise, separated into upper GI complaints (reflux, heartburn, belching, bloating, stomach cramps, vomiting, nausea), lower GI complaints (abdominal pain, side ache, flatulence, urge to defecate, diarrhea, intestinal bleeding, loose stool), and other exercise related complaints (dizziness, muscle cramps, headaches and urge to urinate). All complaints were scored on a 10-point scale from 1 (“no problems”) to 10 (“never been worse”). The corresponding author of this study will share the FFEQ for practical or research purposes upon request.

#### 2.3.3. Post-Race Complaints Questionnaire 

All runners also received a questionnaire about GI complaints and other exercise related complaints (as previously described) that occurred after their finishing time up until 12 h after finishing, which was an exact copy of part E of the FFEQ, as shown in Figure 1. GI complaints were scored on a scale from 1 (“no problems”) to 10 (“never been worse”). All questionnaires were provided in Dutch, an English translation can be provided upon request. 

### 2.4. Data Analysis

All calculations were performed in Excel (2016) and SPSS (IBM SPSS Statistics, version 25, Armonk, NY, USA). Based on their distribution, variables are expressed as median and interquartile range (IQR). Runners’ characteristics are expressed as age (years), speed (km/h), height (cm) and weight (kg). Nutritional intake, expressed per running distance, is expressed as energy consumption (kcal/h), carbohydrates (CHO), protein, fiber intake (g/h) and fluid intake (ml/h). Descriptive results of complaints during the race and complaints post-race are reported (prevalence as a percentage). Sex differences within each distance were analyzed using Mann-Whitney U tests (for energy, CHO, protein, fluid, fat and characteristics) with gender as the group variable. Comparisons of group characteristics and food and fluid intake between all three running distance groups were tested using a Kruskal-Wallis test. Differences between separate groups were calculated using a Mann-Whitney U test. Complaints were categorized as no complaints (score 1), mild complaints (score 2–4) or severe complaints (score 5–10), as described previously by Pfeiffer et al. (1) for all three distances. Spearman correlation coefficients and partial Spearman correlation coefficients with distance as the covariate were calculated including 95% confidence intervals (CI) using Fisher’s Z transformation for all three distances. Eta-squared (η²) was calculated as effect size based on Kruskal-Wallis ‘H’ and Mann-Whitney ‘U’ values and group size (n) based on Fritz et al. (2010) using the following calculator: https://www.psychometrica.de/effect_size.html#nonparametric [29]. Then, the magnitude of the effect size was assessed based on the more familiar Cohen’s d, in which eta-squared below 0.010 indicated no effect and 0.010–0.039 indicated a small effect. Values above 0.06 indicated an intermediate to large effect [30]. All tests were performed with significance set at *p* ≤ 0.05. 

## 3. Results

### 3.1. Characteristics 

All results are described for the total group of runners, with a focus on three separate distances: marathon, 60 km and 120 km. While the median age of the 120 km runners was slightly higher, the groups did not significantly differ in the other variables, as shown in Table 1. Although no difference was recorded in running speed between groups, it should be taken into account that the exercise level of the athletes running 60 or 120 km was higher in comparison to the marathon group, as they generated the same speed over a longer distance. Groups reported a difference in training hours per week (*p* = 0.002). The largest proportion of the marathon runners (52.0%) and 60 km runners (74.4%) trained for 5–9 h a week, and 62.5% of the120 km runners trained for 9–15 h per week.

Environmental conditions differed slightly between events, but all events occurred in winter or early spring. The average race day temperature ranged between 4.4–11.3 °C, with the humidity between 81–100%. The surface conditions for the marathons consisted of a combination of concrete, asphalt and brick roads. The routes of the 60 km and 120 km races were a combination of asphalt, gravel, beach (sand), soil and dune roads. The marathon routes were essentially flat, with an altitude difference of 100 and 200 m mainly due to the combination of climbing multiple sand dunes and dikes surrounding the isle of Texel in the Netherlands (60 and 120 km). 

### 3.2. Gastrointestinal and Other Exercise Related Complaints during Exercise 

GI complaints or other exercise related complaints were reported by 89.3% of all runners, with 49.0% of the runners reporting one or more severe complaints. The absolute reporting of complaints based on descriptive results differed only slightly between distances, as the prevalence was 87.8% in the marathon vs. 90.7% in 60 km and 100% in 120 km runners. GI complaints in the marathon runners, were significant lower (*p* = 0.024) in comparison to the 120 km runners reporting 100%. No significant difference (*p* < 0.126) was found between marathon and 60 km runners and between 60 km and 120 runners. The most common complaints during competition among all runners were urge to urinate (47.7%), muscle cramps (43.6%) and belching (43.6%). Table 2 shows that only four of the total scored complaints differed significantly between distances (flatulence (*p* = 0.020), side ache (*p* = 0.027), dizziness (*p* = 0.005) and diarrhea (*p* = 0.017). Based on the post hoc analysis between the marathon and 60 km, a significant difference was found for side ache (15.3% vs. 34.9%, *p* = 0.009 and a small η^2^ of 0.025). Between the marathon and 120 km, significant differences were found for dizziness (*p* = 0.013 with a small η² of 0.018) and flatulence (*p* = 0.006 and an intermediate η² = −0.048).

Severe complaints (score > 4) were reported by 49.7% of all runners, 50.0% of marathon runners, 41.9% of 60 km runners and 75.0% of 120 km runners, as shown in Table 2, with no significant difference between marathon and 60 km runners. The prevalence of severe complaints showed a high degree of difference between distances for five complaints: belching (*p* = 0.048), bloating (*p* = 0.008), reflux (*p* = 0.009), dizziness (*p* = 0.004) and diarrhea (*p* < 0.001). Based on the post hoc analysis (Table 3) between the marathon and 120 km runners, significant differences were found for bloating (*p* = 0.005 and a small η² of 0.010) and dizziness (*p* = 0.014 and a borderline small η² of 0.009). Although not significant, differences in reporting were seen between the marathon and 120 km for belching (6.1% vs. 25.0%) and reflux (5.1% vs. 25.0%).

### 3.3. Gastrointestinal and Other Exercise Related Complaints after Exercise

Among all runners, 70.6% reported GI or other exercise related complaints after exercise (12 h after finishing) with 28.6% reporting severe complaints. GI complaints after exercise did occur in 72.0% of the marathon runners, this was significantly lower (*p* = 0.025) in comparison to the 120 km runners (87.5%). No significant difference (*p* = 0.670) was found between marathon and 60 km runners (65.1%) and between the 60 km and 120 km runners. Table 2 shows that the most commonly reported complaints during the 12 h after competition were muscle cramps (47.6%), flatulence (19.0%) and bloating (12.7%). The prevalence of complaints after competition differed significantly between distances for three complaints; muscle cramps (0 = 0.025), nausea (*p* = 0.047) and stomach cramps (*p* = 0.012). Based on the post hoc analysis (Table 3), significant differences were found between the marathon and 60 km, with muscle cramps of 30.6% vs. 51.1% (*p* = 0.036 and a small η^2^ of 0.028), between the marathon and 120 km distance with muscle cramps of 30.6% vs. 62.5% (*p* = 0.032 and a small to intermediate η^2^ of 0.039), for nausea of 12.0% vs. 37.5% (*p* = 0.044 and a small η^2^ of 0.018), and stomach cramps of 5.3% vs. 37.5% (*p* = 0.002 and a small η^2^ of 0.026).

The prevalence of severe GI complaints after exercise was 29.4% for the total group, 28.0% for the marathon, 23.3% for the 60 km and 62.5% for the 120 km distance. No significant difference was found for the prevalence of severe GI complaints after exercise between marathon and 60 km runners. As shown in Table 3, the prevalence of two severe complaints differed between marathon and 120 km; for muscle cramps (13.3% vs 50.0%, *p* = 0.009 and a small η^2^ of 0.035) and belching (1.3% vs 25.0%, *p* = 0.001 and a small η^2^ of 0.014).

### 3.4. Relationship between Gastrointestinal Complaints during and after Exercise

The clear relationship between some GI complaints during competition vs. 12 h after competition is shown in Table 4. Most correlations found were moderate to high. Relevant correlations (based on a substantial prevalence of ~10% or more as shown in Table 2) are reported in this section. For the group including all runners, the most profound correlations were muscle cramps (r = 0.44, *p* < 0.001), intestinal cramps (r = 0.38, *p* < 0.001) and the urge to urinate (r = 0.29, *p* < 0.001). The other significant correlations in the aggregated results were reflux, heartburn, nausea, flatulence and the urge to defecate.

For the marathon distance, relevant correlations were seen in complaint prevalence during vs. after competition for flatulence (28.5% during vs. 22.7% after, r = 0.491, *p* < 0.001), stomach cramps (11.2% vs. 5.3%, r = 0.322, *p* = 0.005), nausea (14.2% vs. 12.0%, r = 0.381, *p* = 0.001), intestinal cramps (10.2% vs. 5.3%, r = 0.328, *p* = 0.004), dizziness (9.2% vs 5.3%, r = 0.333, *p* = 0.004), and the urge to urinate (43.8% vs. 10.7%, r = 0.355, *p* = 0.002). A low correlation was found for abdominal pain (14.0% vs. 4.7%, r = 0.25, *p* = 0.031), belching (41.7% vs. 9.3%, r = 0.248, *p* = 0.032) and muscle cramps (44.9% vs. 30.6%, r = 0.427, *p* < 0.001). The prevalence of vomiting complaints during competition had a moderate to high correlation. The prevalence of the complaints for heartburn and diarrhea was low.

For the 60 km group, all reported correlations were based on a prevalence of complaints ≥10%. Strong associations between complaint prevalence during vs. after competition were found for heartburn (11.6% vs. 11.6%, r = 0.778, *p* < 0.001), muscle cramps (46.5% vs. 51.1%. r = 0.537, *p* < 0.001), stomach cramps (18.7% vs. 11.6%, r = 0.610, *p* < 0.001), and the urge to defecate (18.6% vs. 16.3%, r = 0.648, *p* < 0.001). Slightly smaller associations were found for reflux (23.3% vs. 11.6%, r = 0.487, *p* = 0.001), intestinal cramps (23.2% vs. 13.9%, r = 0.404, *p* = 0.007), and the urge to urinate (53.5% vs. 13.9%, r = 0.36, *p* = 0.002). 

The high associations found for the 120 km group were for side ache (r = 1.000, *p* < 0.001), diarrhea (r = 1.000, *p* < 0.001) and headache (r = 1.000, *p* < 0.001). 

### 3.5. Macronutrient Intake

The average energy intake for the total group of runners was 200 (139;291) kcal/h. Average fluid intake was 358 (245;478) ml/h and CHO intake was 42.1 (31.1;63.3 g/h. No differences were seen for the consumption of fat, protein and dietary fiber between distances (*p* > 0.05). The average nutrient consumption per distance ranged between 0.2–1.2 g/h for fat, 1.1–3.0 g/h for protein, and 0.25–0.6 g/h for dietary fiber. There was no sex-effect found within distance groups based on nutrient or fluid intake (*p* > 0.05).

**Figure 2:**
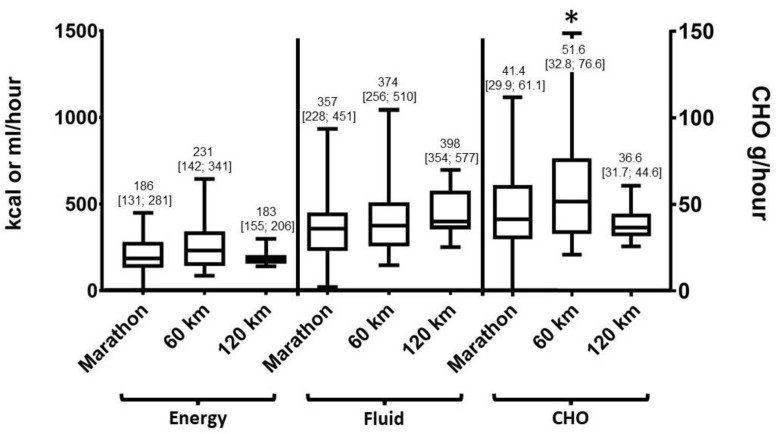
Energy, fluid and CHO intake during competition. The asterisk (*) indicates a significant difference (*p* < 0.05) between the 60 km and marathon distance.

Figure 2 shows the runners’ hourly CHO, fluid and energy intake during competition. A significantly higher CHO intake was seen in the 60 km group vs. the marathon group (*p* = 0.042). No other absolute differences were seen in energy or macronutrient intake between distance groups (*p* > 0.05). The relative macronutrient distribution of CHO during competition showed meaningful descriptive differences between distances: marathon: 88.2%, 60 km: 91.6% and 120 km: 81.7 % of total energy consumed.

Most athletes (77.2%) consumed more than 30 grams of CHO per hour, with small but not significant differences between groups: 74.5% of the marathon runners, 81.4% of 60 km runners and 87.5% of the 120 km runners. Only 27.6% of the marathon runners, 39.5% of the 60 km runners and 12.5% of the 120 km runners exceeded the CHO intake of 60 grams per hour. Overall, 37.8% of the marathon runners, 41.1% of the 60 km runners and 50% of the 120 km runners exceeded a fluid intake of 400 mL/h. Only 4% of the marathon runners, 16.3% of the 60 km runners and 25% of the 120 km runners exceeded a fluid intake of 600 mL/h. 

### 3.6. Correlations between GI Complaints and Macronutrient Intake 

Mainly negative correlations were found in this study, suggesting that those with few or no complaints were able to consume a higher amount of these specific nutrients. Table 5 shows the correlations between GI complaints and macronutrient intake per distance and for all runners together. When all distance groups were combined, four negatively correlated GI complaints were found. Belching was correlated with fiber intake (r = −0.19, *p* = 0.022), abdominal pain with fluid intake (r = −0.17, *p* = 0.042), diarrhea with CHO intake (r = −017, *p* = 0.040) and vomiting with fat intake (r = −0.17, *p* = 0.042). 

Complaints of dizziness at the marathon distance were negatively correlated with energy intake (r = −0.209, *p* = 0.039) and CHO intake (r = −0.199, *p* = 0.049). This data indicated that less dizziness occurred in runners consuming a higher amount of energy and/or CHOs. At the marathon distance, the intake of energy (r = −0.234, *p* = 0.039) and protein (r = −0.201, *p* = 0.048) was negatively correlated to abdominal pain, but the intake of fat (r = 0.215, *p* = 0.033) was positively correlated with abdominal pain. Fat (r = −0.225, *p* = 0.026) and fiber (r = −0.248, *p* = 0.014) consumption were also negatively correlated to belching. A high fat (r = −0.215, *p* = 0.033 and r = −0.243, *p* = 0.016) consumption was also associated with the mild and total amount of symptoms reported, respectively. 

Finally, some positive correlations were also found between urination frequency and intake of energy (r = 0.236), CHO (r = 0.220), fat (r = 0.300), protein (r = 0.206) and fiber (r = 0.316) at the marathon distance (*p* < 0.041). This suggests that a higher intake of energy at this distance resulted in more frequent urination stops. On the other hand, at the 120 km distance the opposite relationship was found between defecation frequency and CHO intake (r = −0.730, *p* = 0.040). Another negative correlation at the 120 km distance was found between fluid (r = −0.764, *p* = 0.027) intake and severe GI complaints, indicating that more complaints occurred when less fluid was consumed. No relation was found between muscle cramps and energy or nutrient intake (*p* < 0.05).

## 4. Discussion

Regardless of distance, the total number of runners experiencing GI complaints was high, both during and 12 h after competition. During competition, the most commonly reported exercise related complaints were the urge to urinate and muscle cramps, while the most reported GI complaints were belching and flatulence. During the 12 h after competition, flatulence and bloating were the most reported GI complaints. Both during and 12 h after competition, muscle cramps were reported by almost half of the running population. Those running longer distances had a higher prevalence of complaints during exercise for 4 out of 16 scored complaints: bloating, side ache, flatulence and dizziness. Positive correlations for self-reported GI complaints during and for the 12 h after competition were moderate to high. 

During this study, we measured the self-reported food intake and GI complaints of runners at marathon, 60 km and 120 km distances. Although previous literature has suggested that the prevalence of GI complaints was mainly the result of distance, which leads to splanchnic hypoperfusion [6], in the current study the severity of GI and other complaints was not generally influenced by distance except for a small number of single complaints. By adding the calculation of an effect size (η²) we were able account for the difference in group sizes, leading to the removal of almost 50% of the originally calculated significant differences between distances. 

The notable outcome of this study is that data showed that the incidence of one or more reported (GI) complaints was high for all distances, and ranged between 88% and 100%. By comparison, previous studies reported only a 50% prevalence of GI complaints in 160 km runners [31] and a 82.9% prevalence of GI complaints in 60 km runners [3]. Again, scores for severe GI complaints were relatively high in the current study (50% at the marathon distance and 41.9% at 60 km) when compared to other studies, such as a 4% prevalence at a marathon distance [1] and a 7.3% prevalence at 60 km [3]. The fact that the absolute prevalence and severity for most GI complaints did not differ between distances suggests that distance itself is not the only factor influencing the prevalence of GI complaints. 

A multifactorial cause of GI complaints is likely [2,6]. Various factors may influence the onset of GI complaints, such as food choices [11], individual fitness [6], the mechanical impact of running, which may be related to the time under tension (distance) and the physiological effect of reduced mesenteric blood shifting toward skin blood flow to cool the body during prolonged exercise [6]. It has been hypothesized that mechanical bouncing damages the gastrointestinal tract [6,32,33]. GI hypoperfusion, caused by stress hormone responses is likely related to intestinal injury, but it has been suggested that sucrose feeding during exercise may lower intestinal injury [34]. While it seems reasonable to suggest gut damage can induce GI complaints., previous studies have not shown a clear relation between gut injury and self-reported GI complaints [5,35]. Notably, GI complaints have been associated with macronutrient intake [1,2,4]. Our study showed comparable results to those previously observed by our lab for mainly negative associations between macronutrient intake and GI complaints in runners, ranging from r: −0.29 to −0.41 [3]. Correlations between GI complaints and macronutrients in this study can be traced to three different patterns. The first is that the consumption of nutrients, such as dietary fat and fiber intake were most often negatively associated with GI complaints. This means that runners scoring high intakes for fat, fiber and protein reported a lower number of complaints, likely due to the fact that they are not experiencing many problems, they are able to consume a higher amount of macronutrient rich foods. The second pattern is that individual pathology may influence correlations, for example, a group that reports very low intake of specific nutrients may still report a high number of complaints. This could be a response to the development of a severe complaint such as diarrhea, resulting in runners who defecate multiple times being less likely to consume high amounts of CHO as this would worsen the existing complaint. Or, these runners changed their intake strategy and behavior before the race based on previous experience because of a prior sensitivity to particular complaints, but due to other mechanisms the complaints developed regardless. Finally, nutrient intake may influence the pattern of urine excretion and defecation, as the high intake of nutrients during the marathon was positively associated with more frequent urinary stops. This is likely because on a cold race day, most runners were using sports drinks as their main energy source, resulting in a higher fluid intake than their actual fluid needs. As a result of this complex relationship between food intake and GI complaints, the causality of the effect of macronutrients inducing GI complaints remains a subject of debate [2,31].

This study adds to our knowledge by presenting questionnaire results that indicate the prevalence of GI complaints during a race and 12 h after race competition. While a crossover design in a controlled lab setting could assess differences between distances, this type of study is difficult to execute. To our knowledge, only one study using a lab-based approached looked at the effect of running on recovery from complaints within a short time frame (1–2 h) after 180 min of running [36]. Another study looked at the prevalence of complaints in various running events in the first 24 h after finishing a race [9]. The participants in the current study reported somewhat similar results to Costa et al., with a reported prevalence of 8–20% in various categories of complaints [36]. These results were much higher than those of Ter Steege et al., who reported up to 4% of different single complaints such as nausea and diarrhea [9]. Although both studies covered exercise times/distances up to the marathon, e.g., the lower time window of endurance running, the Costa study protocol may have been more intensive because of the exercise intensity in combination with a much higher carbohydrate intake. Based on our study results showing moderate to strong correlations found between complaints during and after competition, we suggest that intestinal cramps, diarrhea and muscle cramps may be strong predictors for also having these complaints 12 h after the race. Although the mechanisms may differ (i.e., gastrointestinal damage, reperfusion damage, nutrient malabsorption), the complaint itself was directly related to some type of tissue damage, and therefore substantial healing time is needed. 

Because questionnaires were used for self-reporting of food, fluid intake and GI complaints, the results may be influenced by misreporting [37]. While the reporting quality for this type of questionnaire has not yet been specified, other food frequency questionnaires have shown an underestimation of energy intake by approximately 10% [38]. Given the lack of a more objective method to quantify GI complaints, the use of a questionnaire remains the most accessible and least time-consuming intervention in a research setting. Although continuous observation may seem to be a more accurate way to measure GI complaints, we previously demonstrated that a large reporting difference exists between such observation and recalling GI complaints during competition or when a questionnaire is used after competition [4]. Regardless, self-reporting is subjective and will thus necessarily result in individual reporting differences based on the runner’s personal perceptions; also, we did not measure hydration status during this study. Thus, real correlations may have been influenced by the methods, but it is difficult to determine to what extent.

Study results may have been influenced by several other factors. Runners were included on a voluntary basis, and only the results of those finishing the race were included in our analysis. We do not have a clear understanding of the quantity of symptomatic vs. non-symptomatic runners included in this study, and baseline measurements of complaints present before the start were not obtained. However, it is known that almost all runners reported complaints, therefore it seems to be a natural phenomenon to report at least one or more complaints during the marathon distance or further. Severe gastrointestinal complaints, such as diarrhea or vomiting, could be a reason for not finishing a race and therefore influence the prevalence of these complaints in our results. We expect that runners’ fitness level also influenced the study results, as a higher fitness level is required for 60 or 120 km distances, which has been confirmed by the higher number of training hours reported by these groups. The self-reported complaints were likely influenced by previous (ultra)marathon experiences as the runners were well-experienced and trained to perform in this type of event, resulting in modifications of food and fluid intake during competition. Although we were not able to control for this, we speculate that the small differences in GI complaints found between distances may therefore be indicative of the exercise duration and/or race distance. The cold weather conditions during the races in this study may influence the generalizability of these results. Although hot conditions may trigger a larger thirst response, the sweat rate could be higher as well, therefore the expected body weight differences may be even larger in hot conditions. In combination with pictures, the addition of pre-specified fluid volumes to the questionnaire may have positively influenced the fluid reporting in comparison to the earlier version of this questionnaire. However, we did not assess how pre-race nutrition influenced complaints during the race as start times varied, which made it difficult to standardize assessing the potential relation between both. The time frame for data collection after the race was relatively short (after the finish but before midnight and the complaints during the first 12 h after the finish were reported on the day after the race) and may have influenced the accuracy of the reporting. We hypothesize that the GI or other exercise related complaints reported after competition may be the result of intestinal or respiratory injury that was caused during competition. As it has been difficult to show a clear relationship between gut injury, GI complaints and macronutrient intake during exercise [5], the reporting of complaints after competition may help to identify complaints that were the result of severe gut or respiratory tract and/or muscle damage. Finally, despite the low number of 120 km runners in this article, the study included a high percentage of 120 km runners (38%) in comparison to the marathon (12%) and the 60 km (12%) runners. Due to the nature of the event, the 120 km sample was small relative to the other groups. Therefore, differences and correlations with nutrient intake from other groups were difficult to interpret for the 120 km group, although the small to moderate effect sizes for the differences indicate that a small difference between groups can be expected. 

In conclusion, the prevalence of total reported GI and other exercise related complaints was high at all distances. No more than two to four differences per complaint category (i.e. total complaints vs. severe complaints) were reported between distances. Except for a higher carbohydrate intake in 60 km runners, no differences were found for macronutrient intake. There were small to strong correlations between food and fluid intake and GI complaints without a clear pattern for all distances. Macronutrient intake may influence GI and exercise related complaints, but food choices are likely only one part of the puzzle of factors influencing the occurrence of complaints.

## Figures and Tables

**Table 1:** General characteristics of runners (median and IQR).

Characteristics	Combined Distances	Marathon	60 km	120 km	Significance
	n = 149	n = 98	n = 43	n = 8	*p*-value
Gender (M/F)	119/30	75/23	36/7	8/0	–
Age (years)	43 (36;51)	44 (35;51)	43 (38;53)	47 (41;51)	0.50
Weight (kg)	73 (68;81)	73 (68;81)	74 (69;82)	71 (64.5;73.75)	0.27
Height (cm)	181 (175;185)	180 (173;184)	183 (178;187)	183 (179;185)	0.10
Speed (km/h)	9.9 (9.1;11.3)	10.3 (9.0;11.6)	9.5 (9.1;10.3)	9.6 (9.0;10.3)	0.13
Finish time (hh:mm)	–	4:16 (3:44;4;38)	5:50 (5:10;6:40)	12:30 (11:39;13:20)	–

No significant results existed between groups using a Kruskal-Wallis test with *p*-value set at *p* ≤ 0.05.

**Table 2:** Prevalence of gastrointestinal and exercise related complaints during and after competition.

		Complaints During				Complaints After			
Type of Complaint	Category	Marathon n = 98	60 km n = 43	120 km n = 8	*p*-Value	Marathon N = 75	60 km n = 43	120 km n = 8	*p*-Value
**Upper GI complaints**									
Belching	Total	41.7%	44.2%	62.5%	0.53	9.3%	14.0%	25.0%	0.30
	Severe	6.1%	2.3%	25.0%	0.048^C^	1.3%	0.0%	25.0%	0.000^C^
Bloating	Total	20.4%	20.9%	50.0%	0.15	12.0%	11.6%	25.0%	0.57
	Severe	3.1%	2.3%	25.0%	0.008^C^	0.0%	2.3%	12.5%	0.38
Reflux	Total	17.3%	23.3%	37.5%	0.33	2.7%	11.6%	12.5%	0.12
	Severe	5.1%	0.0%	25.0%	0.009^C^	0.0%	0.0%	12.5%	0.001^C^
Nausea	Total	14.2%	25.6%	37.5%	0.11	12.0%	7.0%	37.5%	0.047^C^
	Severe	2.0%	4.7%	12.5%	0.25	1.3%	2.3%	12.5%	0.15
Stomach cramps	Total	11.2%	18.7%	25.0%	0.34	5.3%	11.6%	37.5%	0.012^C^
	Severe	6.1%	4.7%	12.5%	0.69	2.7%	2.3%	12.5%	0.30
Heartburn	Total	4.1%	11.6%	12.5%	0.21	2.6%	11.6%	0.0%	0.10
	Severe	1.0%	0.0%	0.0%	0.77	1.3%	2.3%	0.0%	0.86
Vomiting	Total	1.0%	4.7%	0.0%	0.34	2.6%	0.0%	0.0%	0.50
	Severe	1.0%	0.0%	0.0%	0.77	1.3%	0.0%	0.0%	0.71
**Lower GI complaints**									
Flatulence	Total	28.5%	39.5%	75.0%	0.020^C^	22.7%	16.3%	50.0%	0.11
	Severe	7.1%	9.3%	25.0%	0.23	6.7%	2.3%	12.5%	0.42
Side ache	Total	15.3%	34.9%	12.5%	0.027^A^	2.7%	0.0%	12.5%	0.10
	Severe	4.1%	11.6%	12.5%	0.21	2.7%	0.0%	12.5%	0.10
Urge to defecate	Total	13.3%	18.6%	37.5%	0.18	6.7%	16.3%	12.5%	0.27
	Severe	5.1%	9.3%	25.0%	0.10	2.7%	2.3%	0.0%	0.90
Intestinal cramps	Total	10.2%	23.2%	25.0%	0.10	5.3%	13.9%	25.0%	0.11
	Severe	4.1%	2.3%	12.5%	0.41	4.0%	2.3%	12.5%	0.40
Abdominal pain	Total	10.2%	14.0%	25.0%	0.42	6.6%	4.7%	0.0%	0.698
	Severe	4.1%	0.0%	12.5%	0.16	1.3%	0.0%	0.0%	0.712
Diarrhea	Total	1.0%	0.0%	12.5%	0.017^C^	6.6%	7.0%	12.5%	0.80
	Severe	0.0%	0.0%	12.5%	0.000^C^	1.3%	2.3%	0.0%	0.15
Loose stool	Total	1.0%	2.3%	0.0%	0.78	1.3%	0.0%	0.0%	0.71
	Severe	1.0%	2.3%	0.0%	0.78	0.0%	0.0%	0.0%	1.00
**Other exercise related complaints**									
Muscle cramps	Total	44.9%	46.5%	12.5%	0.19	30.6%	51.1%	62.5%	0.025^AC^
	Severe	21.4%	20.9%	12.5%	0.84	13.3%	20.9%	50.0%	0.034^C^
Urge to urinate	Total	43.8%	53.5%	62.5%	0.40	10.7%	13.9%	12.5%	0.89
	Severe	18.4%	7.0%	12.5%	0.21	4.0%	2.3%	0.0%	0.77
Headache	Total	11.2%	7.0%	12.5%	0.72	16.3%	7.0%	12.5%	0.13
	Severe	2.0%	0.0%	0.0%	0.59	2.0%	2.3%	0.0%	0.90
Dizziness	Total	9.2%	2.3%	37.5%	0.005^C^	5.3%	7.0%	25.0%	0.11
	Severe	4.1%	0.0%	25.0%	0.004^C^	1.3%	0.0%	12.5%	0.034*

Ranked from high to low for prevalence during marathon running, none of the runners reported intestinal bleeding. * Significant difference using a Kruskal-Wallis test (*p* ≤ 0.05). When the Mann-Whitney U test was significant, the * for the Kruskal-Wallis test was over-ruled, with *p*-value set on ≤0.05. A stands for a difference between marathon and 60 km, B for a difference between 60 km and 120 km, and C for a difference between marathon and the 120 km distance.

**Table 3:** Significant *p*-values and effect sizes (η²) based on prevalence of GI and exercise related complaints during and after competition (Table 2).

		Complaints During			Complaints After		
Type of Complaint	Category	Marathon vs. 60 km (A)	60 km vs. 120 km (B)	Marathon vs. 120 km (C)	Marathon vs. 60 km (A)	60 km vs. 120 km (B)	Marathon vs. 120 km (C)
**Upper GI complaints**							
Belching	Total	–	–	–	–	–	–
	Severe	–	–	η² = −0.007, *p* = 0.001	–	–	η² = −0.014, *p* = 0.001
Bloating	Total	–	–	–	–	–	–
	Severe	–	–	η² = −0.010, *p* = 0.005	–	–	–
Reflux	Total	–	–	–	–	–	–
	Severe	–	–	η² = −0.008, *p* = 0.030	–	–	η² = −0.004, *p* = 0.002
Nausea	Total	–	–	–	–	–	η² = −0.018, *p* = 0.044
	Severe	–	–	–	–	–	–
Stomach cramps	Total	–	–	–	–	–	η² = −0.026, *p* = 0.002
	Severe	–	–	–	–	–	–
**Lower GI complaints**							
Flatulence	Total	–	–	η² = −0.048, *p* = 0.006	–	–	–
	Severe	–	–	–	–	–	–
Side ache	Total	η² = −0.025, *p* = 0.009	–	–	–	–	–
	Severe	–	–	–	–	–	–
Diarrhea	Total	–	–	η² = −0.003, *p* = 0.021	–	–	–
	Severe	–	–	η² = −0.003, *p* < 0.001	–	–	–
**Other exercise related complaints**							
Muscle cramps	Total	–	–	–	η² = −0.028, *p* = 0.036	–	η² = −0.039, *p* = 0.032
	Severe	–	–	–	–	–	η² = −0.035, *p* = 0.009
Dizziness	Total	–	–	η² = −0.018, *p* = 0.013	–	–	–
	Severe	–	–	η² = −0.009, *p* = 0.014	–	–	–

Ranked from high to low for prevalence during marathon running. Cursive values were not reported as actual differences as results of the small effect size η². No significant difference was found for urge to urinate, urge to defecate, intestinal cramps, loose stool and vomiting.

**Table 4:** Correlations (r) and 95% CI between complaints during and after exercise.

Type of Complaint	All Distances (n = 149)	Marathon (n = 98)	60 km (n = 43)	120 km (n = 8)
**Upper GI complaints**				
Belching	–	0.25 (*p* = 0.032) (0.05:0.43)	–	–
Reflux	0.36 (*p* < 0.001) (0.21:0.49)	–	0.49 (*p* = 0.001) (0.22:0.69)	–
Nausea	0.36 (*p* < 0.001) (0.21:0.49)	0.38 (*p* = 0.001) (0.60:0.80)	–	–
Stomach cramps	–	0.32 (*p* = 0.005) (0.13:0.49)	0.61 (*p* < 0.001) (0.38:0.77)	–
Vomiting	0.42 (*p* < 0.001) (0.28:0.54)	0.71 (*p* < 0.001) (0.60:0.80)	–	–
**Lower GI complaints**				
Flatulence	0.43 (*p* < 0.001) (0.29:0.55)	0.49 (*p* < 0.001) (0.32:0.63)	–	–
Side ache	–	–	–	1.00 (*p* < 0.001) (1.00:1.00)
Urge to defecate	0.35 (*p* < 0.001) (0.20:0.49)	–	0.65 (*p* < 0.001) (0.43:0.79)	–
Intestinal cramps	0.38 (*p* < 0.001) (0.23:0.51)	0.33 (*p* = 0.004) (0.14:0.49)	0.40 (*p* = 0.007) (0.12:0.63)	–
Abdominal pain	–	0.25 (*p* = 0.031) (0.05:0.43)	–	–
Diarrhea	0.46 (*p* < 0.001) (0.33:0.58)	0.43 (*p* < 0.001) (0.25:0.58)	–	1.00 (*p* < 0.001) (1.00:1.00)
**Other exercise related complaints**				
Muscle cramps	0.44 (*p* < 0.001) (0.30:0.56)	0.43 (*p* < 0.001) (0.25:0.58)	0.54 (*p* < 0.001) (0.28:0.72)	–
Urge to urinate	0.29 (*p* = 0.001) (0.13:0.43)	0.36 (*p* = 0.002) (0.17:0.52)	–	–
Headache	–	–	–	1.00 (*p* < 0.001) (1.00:1.00)
Dizziness	–	0.33 (*p* = 0.004) (0.14:0.50)	–	–

No correlation was found for bloating, heartburn, loose stool and intestinal bleeding. Data for the group of all distances were based on Spearman Partial Correlations with distance as the covariate. Data for separate distances groups were based on Spearman Correlations.

**Table 5:** Correlations and 95% CI between complaints and macronutrient intake.

Type of Complaint	All Distances (n = 149)	Marathon (n = 98)	60 km (n = 43)	120 km (n = 8)
**Upper GI complaints**				
Belching	−0.19 Fiber/h (*p* = 0.022) (0.03:0.34)	−0.23 Fat/h (*p* = 0.026) (−0.41:−0.03) −0.25 Fiber/h (*p* = 0.014) (−0.43:−0.05)	–	–
Reflux	–	–	0.31 Fiber/h (*p* = 0.042) (0.01:0.56)	–
Vomiting	−0.17 Fat/h (*p* = 0.042) (0.01:0.32)	–	–	–
**Lower GI complaints**				
Urge to defecate	–	–	–	-0.78 CHO/h (*p* = 0.021) (−0.96:−0.18)
Intestinal cramps	–	–	–	0.77 PRO/h (*p* = 0.026) (0.14:0.96)
Abdominal pain	−0.17 Fluid/h (*p* = 0.042) (−0.32:−0.01)	−0.23 Kcal/h (*p* = 0.039) (−0.41:−0.04) −0.20 PRO/h (*p* = 0.048) (−0.38:−0.01) 0.22 Fat/h (*p* = 0.033) (−0.40:−0.02)	–	–
Diarrhea	−0.17 CHO/h (*p* = 0.040) (−0.32:−0.01)	–	–	–
**Other exercise related complaints**				
Urge to urinate	–	–	0.33 Kcal/h (*p* = 0.030) (0.04:0.58) 0.34 CHO/h (*p* = 0.028) (0.04:0.58)	–
Dizziness	–	−0.21 Kcal/h (*p* = 0.039) (−0.40:−0.02) −0.20 CHO/h (*p* = 0.049) (−0.39:−0.01)	–	–

No correlation was found for energy or macronutrients and heartburn, stomach cramps, vomiting, nausea, flatulence, diarrhea, headache and muscle cramps. Data for the group of all distances were based on Spearman Partial Correlations with distance as the covariate. Data for separate distance groups were based on Spearman Correlations.
